# Comparison of the Effectiveness of Smart Vision Glasses Combined With a White Cane Versus a White Cane Alone for Indoor and Outdoor Mobility in Blind Students

**DOI:** 10.7759/cureus.114262

**Published:** 2026-08-10

**Authors:** Parikshit Gogate, Sayantani Ghosh, Mohankumar Naikodi, Supriya Phadke, Apoorva Shitole, Nayan Bajantri, Susmita Banerjee, Veshal Madan

**Affiliations:** 1 Ophthalmology, Dr. D. Y. Patil Vidyapeeth, Pune, IND; 2 Optometry, Dr. D. Y. Patil Institute of Optometry and Visual Sciences, Pune, IND; 3 Optometry, Community Eye Care Foundation, Pune, IND; 4 Ophthalmology, Community Eye Care Foundation, Pune, IND; 5 Research, Smart Health Technologies Pvt. Ltd., Bengaluru, IND; 6 Optometry and Visual Sciences, Dr. D. Y. Patil Institute of Optometry and Visual Sciences, Pune, IND

**Keywords:** assistive technology, audio-based navigation, blindness, mobility, navigation, smart vision glasses, visual impairment, white cane

## Abstract

Background: Blind individuals face mobility challenges regularly, and audio-based smart vision glasses (SVGs) may enhance safe, independent navigation by providing real-time voice guidance beyond a white cane in mobility.

Objective: The objective of the study is to evaluate the effectiveness of an audio-based SVG system in improving navigation performance among individuals with low vision when used in combination with a white cane.

Methods: A within-subject crossover study compared white cane navigation with and without the audio-based SVG Premium (by SHG Technologies Pvt. Ltd., Bengaluru, India) among students with blindness. Each student completed two trials on both the indoor and outdoor mobility routes under two experimental conditions: (A) using a white cane alone and (B) using a white cane in combination with SVGs. The indoor mobility assessment was conducted on a 100 m trial designed within the school premises. The outdoor assessment was performed on a 410 m outdoor trail. Observers recorded the primary outcomes (completion time, obstacle collision, navigation mistakes, stops, and assistance requests) and assessed ease of navigation and confidence on a 5-point scale in which 0 is no difficulty or lowest intensity and 5 is most difficult or highest intensity after each trial. Paired statistical tests were used to compare performance with the cane and SVG to that with the cane alone.

Results: A total of 29 students were enrolled in the study, comprising 21 male students (72.4%). The mean age of the students was 15.1 ± 2 years (range: 10-20 years), and students with clinically proven blindness were recruited in the study from two schools for the blind: Miraj (National Association for the Blind (NAB) Miraj) and Pune (Patashibai Lunkad School for the Blind). All subjects had bilateral blindness, best-corrected visual acuity < 1/60 in the better eye, but retained hearing and cognitive capacity to follow instructions. In the indoor navigation task, the audio-based SVG system significantly improved several aspects of mobility performance compared with the white cane alone. SVG significantly reduced collisions, hesitations, and total navigation errors while improving ease of navigation and user confidence (all p < 0.05). Indoor collisions decreased from 4.28 ± 2.72 to 2.00 ± 1.83, and outdoor collisions decreased from 1.62 ± 1.47 to 0.62 ± 1.02. Total navigation errors were reduced from 7.24 ± 3.50 to 3.79 ± 2.09 indoors and from 4.10 ± 2.34 to 2.03 ± 1.64 outdoors. Participants reported significantly greater ease of navigation and confidence with SVG. Completion time increased significantly in both indoor and outdoor tasks (p < 0.001).

Conclusion: The findings suggest that audio-based SVG technology can significantly enhance mobility performance and user confidence among individuals with low vision. Such wearable assistive devices have the potential to improve independence, safety, and quality of life, particularly in resource-constrained settings where access to advanced rehabilitation services may be limited.

## Introduction

Visual impairment remains a major global public health challenge, affecting more than 2.2 billion people worldwide, with a disproportionate burden in low- and middle-income countries [1,2]. Blindness is defined as visual acuity worse than 3/60 (20/400) in the better eye with the best possible correction or a visual field constricted to less than 10° from fixation [2]. Individuals with low vision experience significant functional limitations that cannot be fully corrected with spectacles, affecting essential activities such as reading, recognizing faces, walking, and independent navigation [3].

In India, visual impairment is a leading cause of disability, affecting approximately 13 million people, while earlier national surveys reported a low-vision prevalence of 0.27%, with higher rates in rural populations [1,2]. Access to low-vision rehabilitation and assistive technologies remains limited, creating a substantial gap between clinical need and service availability [4]. Conventional mobility aids, particularly the white cane, are widely used for independent travel; however, they primarily detect ground-level obstacles and provide limited information regarding distant hazards, spatial orientation, or dynamic environments. Guide dogs are effective but require extensive training, are costly, and remain largely inaccessible in developing countries. Consequently, many individuals with visual impairment continue to rely on caregivers, reducing independence and social participation [5,6].

Recent advances in wearable assistive technology have introduced smart vision glasses (SVGs) as promising tools for improving mobility and environmental awareness. These devices integrate cameras, ultrasonic sensors, computer vision algorithms, Bluetooth connectivity, and audio feedback systems to translate visual information into spoken instructions or haptic cues, enabling users to detect obstacles and navigate indoor and outdoor environments more safely [5,7,8]. Several navigation systems combining Global Positioning System (GPS), Bluetooth Low Energy, Internet of Things (IoT) technologies, and artificial intelligence (AI) have demonstrated improvements in obstacle detection, spatial orientation, and user confidence [6,7,9]. Nevertheless, most published studies have focused on prototype development or laboratory-based evaluations, with limited evidence regarding the functional effectiveness of commercially available SVGs in real-world settings, particularly among individuals with low vision in India [8].

Therefore, the present study aimed to evaluate the effectiveness of SVGs in improving indoor and outdoor mobility, obstacle avoidance, navigation performance, and user confidence among individuals with visual impairment. The findings may provide evidence for integrating wearable assistive technologies into low-vision rehabilitation services to enhance independent mobility and quality of life.

## Materials and methods

A within-subject crossover experimental study was conducted among blind students from two schools for the blind in Maharashtra, India. Ethical approval was obtained from the Institutional Ethics Committee of Dr. D. Y. Patil Institute of Optometry and Visual Sciences, Dr. D. Y. Patil Vidyapeeth, Pune (Ref. No. DYPIOVS/CO/78F/2025). Students completed navigation tasks under two conditions: using a white cane alone and a white cane combined with audio-based SVGs. Data were analyzed using the paired t-test, with statistical significance set at p < 0.05.

We selected students diagnosed with blindness (visual acuity less than 1/60 to blind) who were able to understand and follow audio instructions, aged >10 years, and who were willing to participate. Consent was given by the class teacher and principal of the school. Students who had hearing impairment that would affect their ability to use audio-based systems, cognitive impairments that limit their ability to follow navigation instructions, and any other mobility-related disability (e.g., motor disabilities) were excluded from the study.

Throughout the experiment, each student utilized their own standardized white mobility cane. Students who were in the aided condition wore a lightweight audio-based “SVGs Premium” by SHG Technologies Pvt. Ltd., Bengaluru, India (a spectacle-mounted navigation aid). The gadget has a front-facing camera, a Light Detection and Ranging (LiDAR) range sensor, and an audio interface. This non-invasive device, installed on a spectacle frame, gives real-time verbal prompts via a Bluetooth earpiece. Obstacle identification and wayfinding help are among the SVGs’ functions: during navigation, they proclaim obstacle and distance information via audio. Students were trained on the equipment (calibrating the sensors, manipulating the controls, and interpreting verbal commands) until they exhibited competency. Before each experiment, all devices were tested for battery and operation (flashlight, camera, LiDAR, and speaker). Observers recorded any equipment problems that occurred during the trial.

A within-subjects crossover design was implemented. Each participant did two trials of each route (indoor and outdoor) under two conditions: (A) white cane alone and (B) white cane with SVGs. Observers who recorded the trials were blinded to the condition sequence and had no idea which mode was tried first. Before testing, students underwent a standardized 30-90-minute training session on how to use the SVGs. This session comprised a demonstration, hands-on training using controls (braille-coded buttons), and at least one practice walkthrough of a simulated route. A rest period was provided between condition trials to reduce tiredness.

Indoor course

A planned 100 m course was arranged within the school. The walkway began at the classroom door and proceeded to the canteen (~50 m), the dormitory gate (~30 m), the school stage (~10 m), and ultimately back to the classroom. The corridor and open sections had usual barriers (e.g., plastic chairs and potted plants) as well as occasional stationary persons to imitate daily bustle. The illumination was standard classroom lighting, and traffic along the path was low. Students began at the classroom door and were directed down the defined corridor path via the cafeteria area, past the dormitory gate, to the stage and the back. Initially, students utilized only a white cane, the standard navigation approach. Later, they were introduced to SVG and received real-time voice suggestions. During each walk, the observer measured completion time and recorded mistakes using a predefined checklist. A mistake was defined as any obstacle, collision, or missed turn; a collision occurred when the student body or cane obstructed. When the individual stopped moving for more than 5 s, a hesitation was recorded. Observers took note of any pleas for aid or instruction as well as any symptoms of worry or exhaustion.

Outdoor course

The 410 m outdoor circuit started at the school entrance, traveled 20 m straight, 50 m to the institute's main gate, 135 m to the main road, and returned to the start. The external environment consisted of high curbs, uneven pavement, pedestrian activity, and ambient noise. Trials were held in clear weather to minimize rain and glare, and observers documented any unexpected environmental circumstances. Students travelled from the school gate to the institute's entrance gate and the main road loop under regular daylight circumstances. The same definitions of mistakes, collisions, and hesitations were used. Because ambient noise and traffic were higher, observers remained in close proximity to students without providing any cues and observed any environmental interference that happened throughout each walk.

Statistical analysis was done using various statistical tests. Continuous variables (completion time) were compared using paired Student's t-test. Count variables (number of collisions, hesitations, and total navigation errors) were analyzed using the Wilcoxon signed-rank test. Ordinal variables obtained from the 5-point Likert scales (ease of navigation, confidence, anxiety, comfort, additional feedback, and device function) were also compared using the Wilcoxon signed-rank test. Binary outcomes (obstacle collisions and requests for assistance, recorded as present or absent during each trial) were analyzed using McNemar's test for paired categorical data.

Data collection

Each performance was judged based on grading, in which 0 is no difficulty and 5 is the most difficult or highest intensity. Navigation performance was assessed using objective, subjective, and observational measures. Completion time (seconds) was recorded from the start to the finish of each route and served as an indicator of navigation efficiency, with shorter times reflecting better performance. Obstacle collisions were defined as any physical contact with objects such as furniture, plants, walls, or door frames; fewer collisions indicated safer navigation. Hesitations were recorded when students paused for more than 5 s to reassess their route, reflecting uncertainty or increased cognitive effort. Total navigation errors included collisions, incorrect turns, missed turns, and significant route deviations, providing an overall measure of navigational accuracy.

Assistance requests were documented whenever the student sought verbal guidance or clarification during navigation. After each trial, students rated ease of navigation and confidence on separate 5-point Likert scales, with higher scores indicating greater ease and confidence. These measures captured students' perceptions of task difficulty and trust in the navigation system.

Observers also recorded the presence or absence of anxiety or fatigue, based on visible signs such as facial tension, rapid breathing, slowed walking, or exhaustion. Physical comfort was assessed through students’ feedback and observable signs of discomfort associated with device use. Any device glitches, including audio interruptions, inaccurate instructions, or system failures, were documented. Finally, students identified their preferred navigation method (white cane alone or white cane with the SVG system), providing insight into user acceptance and potential real-world adoption.

## Results

A total of 29 students were enrolled in the study, comprising 21 male students (72.4%). The mean age of the students was 15.1 ± 2 years (range: 10-20 years), and students with clinically proven blindness were recruited in the study from two schools for the blind in Miraj (National Association for the Blind, Miraj) and Pune (Patashibai Lunkad School for the Blind). All subjects had bilateral blindness (best-corrected visual acuity < 1/60 in the better eye, under WHO standards) but retained hearing and cognitive capacity to follow instructions. In the indoor navigation task, the audio-based SVG system significantly improved several aspects of mobility performance compared with the white cane alone.

Table 1 shows the results of navigation in the indoor trial comparing the Cane with Cane + SVG.

**Table 1:** Results of navigation in indoor trial comparing Cane with Cane + SVG. SVG: smart vision glass

Indoor setting	Cane	Cane + SVG	p-value
Number of collisions	4.3 ± 2.7	2.0 ± 1.8	<0.001
Number of hesitations	3.0 ± 1.7	1.7 ± 1.0	<0.001
Total number of errors	7.2 ± 3.5	3.8 ± 2.0	<0.001
Number of obstacle collisions	0.1 ± 0.3	0.3 ± 0.5	0.096
Number of requests for assistance	0.17 ± 0.6	0.1 ± 0.4	0.161
Completion time in seconds	229.2 ± 33.1	254.8 ± 39.1	<0.001
Ease of navigation (1-5 Likert scale)	3.8 ± 0.8	4.6 ± 0.6	<0.001
Confidence (1-5 Likert scale)	4.0 ± 1.00	4.7 ± 0.6	<0.001
Anxiety (1-5 Likert scale)	0.9 ± 0.1	1.0 ± 0.0	0.326
Comfort issues (1-5 Likert scale)	1.0 ± 0.0	1.0 ± 0.0	<0.001
Additional feedback	1.0 ± 0.0	1.6 ± 1.2	0.008
Device function (1-5 Likert scale)	0.6 ± 0.5	0.7 ± 0.7	0.162

No significant differences were observed for obstacle collisions recorded as binary events (p = 0.096), assistance requests (p = 0.161), anxiety/fatigue (p = 0.326), or device glitches (p = 0.163). No participant reported physical discomfort in either condition. Overall, the findings suggest that the SVG system enhanced navigation accuracy, safety, ease, and confidence during indoor mobility, although at the expense of slightly longer route completion times.

Table 2 shows the results of various parameters on the outdoor navigation trail comparing Cane and Cane + SVG.

**Table 2:** Results of various parameters on outdoor navigation trail comparing Cane and Cane + SVG. SVG: smart vision glass

Outdoor setting	Cane	Cane + SVG	p-value
Number of collisions	1.6 ± 1.5	0.6 ± 1.0	<0.001
Number of hesitations	2.5 ± 1.6	1.4 ± 1.0	<0.001
Total number of errors	4.1 ± 2.3	2.0 ± 1.6	<0.001
Number of obstacle collisions	0.1 ± 0.3	0.6 ± 0.5	<0.001
Number of requests for assistance	0.5 ± 1.0	0.2 ± 0.5	0.073
Completion time in seconds	578.0 ± 83.0	626.3 ± 87.0	<0.001
Ease of navigation (1-5 Likert scale)	3.9 ± 0.8	4.6 ± 0.6	<0.001
Confidence (1-5 Likert scale)	4.0 ± 0.9	4.6 ± 0.6	0.001
Anxiety (1-5 Likert scale)	0.9 ± 0.2	0.9 ± 0.3	0.424
Comfort issues (1-5 Likert scale)	1.0 ± 0.0	0.9 ± 0.3	0.043
Additional feedback	1.0 ± 0.0	1.724 ± 1.623	0.023
Device function (1-5 Likert scale)	1.0 ± 0.0	0.9 ± 0.3	0.043

Students experienced significantly fewer collisions when using the SVG (0.62 ± 1.02) than when using the cane alone (1.62 ± 1.47), indicating enhanced obstacle avoidance in outdoor environments (t = 7.616, p < 0.001). Hesitations were also significantly reduced from 2.48 ± 1.64 to 1.41 ± 1.05 (t = 4.950, p < 0.001), suggesting smoother and more confident navigation.

Similarly, total navigation errors decreased significantly from 4.10 ± 2.34 in the cane-only condition to 2.03 ± 1.64 with SVG assistance (t = 9.112, p < 0.001), reflecting improved route-following accuracy. Students reported significantly greater ease of navigation (4.66 ± 0.61 vs. 3.86 ± 0.79; t = -5.883, p < 0.001) and higher confidence levels (4.59 ± 0.63 vs. 4.00 ± 0.85; t = -3.642, p = 0.001) while using the SVG system.

Completion time was significantly longer in the SVG condition (626.3 ± 87.0 s) than in the cane-only condition (578.0 ± 83.0 s) (t = 6.671, p < 0.001), suggesting that students adopted a slower but potentially safer navigation strategy while following auditory guidance. No significant difference was observed in assistance requests (p = 0.073) or observed anxiety/fatigue (p = 0.424). Although statistically significant differences were reported for physical comfort (p = 0.043) and device function (p = 0.043), no participant reported substantial discomfort, and all recorded device glitches were minor, temporary, and resolved without compromising safety.

Overall, the outdoor findings support the effectiveness of the SVG system in improving navigation accuracy, reducing errors and hesitations, and increasing users’ confidence and perceived ease of navigation, although these benefits were accompanied by longer route completion times.

## Discussion

Overall, both indoor and outdoor findings reveal that audio-based SVGs greatly enhanced navigational performance (faster timing, fewer mistakes and collisions) and user confidence in most metrics, with only minor comfort and technical concerns. These findings support previous indications that audio navigation devices might improve mobility and minimize mistakes for blind individuals.

The results of this study are consistent with previous studies on aided navigation. For example, Abidi et al. discovered that contemporary wearable navigation systems, particularly those that combine sensors, smartphone interfaces, and feedback modalities, significantly increase users’ capacity to recognize hazards and travel safety. Our findings also revealed that users obtained more efficient travel and fewer orientation mistakes with auditory guidance, corroborating Abidi et al. [9]. Similarly, Isazade stated that developments in navigation technology (such as mobile applications and voice assistance) considerably improve the quality of life and independence of visually impaired people [10]. Early research, e.g., Gupta et al., showed that audio signals can successfully assist blind or low-vision travelers, and our work adds empirical support to that hypothesis [5].

Our findings highlight the importance of sensory replacement and auditory spatial mapping in assisted navigation. According to Isazade, blind persons frequently orient themselves using auditory cues (such as echoes and ambient sounds) [10]. The SVG technology effectively formalizes this natural technique by providing accurate 3D audio cues and speech direction in real time. This demonstrates that persons with impaired vision may use technical aural input in conjunction with their intrinsic orienting skills to traverse situations more securely. The study emphasizes essential design considerations: the audio interface must be clear, context-aware, and flexible to user preferences. We found that users benefited the most when instructions were timely and uncluttered, which aligned with the criteria of good assistive user interface (UI) design as discussed by Abidi et al. [9].

Unlike lab-only tests, the trial in a classroom setting demonstrated that the device operates well under realistic situations. There have been studies on helping schools for blind students earlier, but they focused on students who had residual visual acuity better than 1/60 [11-13]. Those who had poorer vision could be helped only by rehabilitation. SVG with a cane would greatly increase the safety and efficacy of navigation and thus allow independent mobility to blind persons. Lack of mobility may lead to isolation and resultant anxiety and depression in blind individuals [14]. SVG with a cane would go a long way to prevent that.

This is consistent with Udayakumar et al., who found that cost-effective AI-powered glasses with voice interfaces were highly usable in a diverse Indian cohort [8]. Both their study and ours observed great user engagement with reading and environmental-scanning features, although dynamic “walking aids” signals were employed less frequently, indicating that dynamic guiding requires additional improvement. The instrument would also help blind individuals read and access printed material and greatly increase their social and economic options.

Limitation

Any discrepancies, such as reduced use of the walking-assistance feature, indicate opportunities for design improvement. Our findings contribute to the field by giving data from a low-resource situation, suggesting that audio-based navigation is effective even outside of high-tech facilities.

Although the present study demonstrated promising results for the use of SVG in enhancing mobility among blind students, several limitations should be acknowledged. From a technical perspective, occasional audio dropouts and minor tracking delays were observed during testing, indicating the need for improved system reliability and faster processing capabilities. Future developments should focus on enhancing obstacle-detection algorithms, expanding sensor coverage, improving battery life, and optimizing performance in challenging environments like low-light conditions and noisy outdoor settings. Simplified controls and more intuitive interfaces may further improve usability.

The sample size was relatively small, and students were recruited from only two special schools. Future research should include larger, more diverse populations and evaluate the system in dynamic environments involving road crossings, moving vehicles, crowded public spaces, and night-time navigation. Longitudinal studies are also needed to examine long-term effectiveness and user adaptation.

From a user perspective, students require training before using the device, suggesting a need for more user-friendly designs. Customizable auditory feedback, adjustable volume and speech rates, and reduced cognitive load may improve comfort, accessibility, and acceptance. Addressing these limitations will support the development of more robust, adaptable, and widely applicable audio-based navigation systems for blind people.

## Conclusions

This study demonstrates that audio-based SVGs can significantly improve mobility performance among blind individuals. Compared with using a white cane alone, students experienced fewer collisions, hesitations, and navigation errors, while reporting greater confidence and ease of navigation in both indoor and outdoor environments. Although completion times were longer with the SVG system, the improvement in safety and navigation accuracy suggests a more cautious and effective travel strategy. These findings highlight the potential of affordable audio-based assistive technologies to enhance independence, safety, and quality of life for blind people.
